# On Dorsal Prothoracic Appendages in Treehoppers (Hemiptera: Membracidae) and the Nature of Morphological Evidence

**DOI:** 10.1371/journal.pone.0030137

**Published:** 2012-01-17

**Authors:** István Mikó, Frank Friedrich, Matthew J. Yoder, Heather M. Hines, Lewis L. Deitz, Matthew A. Bertone, Katja C. Seltmann, Matthew S. Wallace, Andrew R. Deans

**Affiliations:** 1 Insect Museum, Department of Entomology, North Carolina State University, Raleigh, North Carolina, United States of America; 2 Biocenter Grindel and Zoological Museum Hamburg, Hamburg University, Hamburg, Germany; 3 Department of Genetics, North Carolina State University, Raleigh, North Carolina, United States of America; 4 Biological Sciences, East Stroudsburg State University, East Stroudsburg, Pennsylvania, United States of America; Field Museum of Natural History, United States of America

## Abstract

A spectacular hypothesis was published recently, which suggested that the “*helmet*” (a dorsal thoracic sclerite that obscures most of the body) of treehoppers (Insecta: Hemiptera: Membracidae) is connected to the 1st thoracic segment (T1; prothorax) via a jointed articulation and therefore was a true appendage. Furthermore, the “*helmet*” was interpreted to share multiple characteristics with wings, which in extant pterygote insects are present only on the 2nd (T2) and 3rd (T3) thoracic segments. In this context, the “*helmet*” could be considered an evolutionary novelty. Although multiple lines of morphological evidence putatively supported the “*helmet*”-wing homology, the relationship of the “*helmet*” to other thoracic sclerites and muscles remained unclear. Our observations of exemplar thoraces of 10 hemipteran families reveal multiple misinterpretations relevant to the “*helmet*”-wing homology hypothesis as originally conceived: 1) the “*helmet*” actually represents T1 (excluding the fore legs); 2) the “*T1 tergum*” is actually the anterior dorsal area of T2; 3) the putative articulation between the “*helmet*” and T1 is actually the articulation between T1 and T2. We conclude that there is no dorsal, articulated appendage on the membracid T1. Although the posterior, flattened, cuticular evagination (PFE) of the membracid T1 does share structural and genetic attributes with wings, the PFE is actually widely distributed across Hemiptera. Hence, the presence of this structure in Membracidae is not an evolutionary novelty for this clade. We discuss this new interpretation of the membracid T1 and the challenges of interpreting and representing morphological data more broadly. We acknowledge that the lack of data standards for morphology is a contributing factor to misinterpreted results and offer an example for how one can reduce ambiguity in morphology by referencing anatomical concepts in published ontologies.

## Introduction

**Figure 1:**
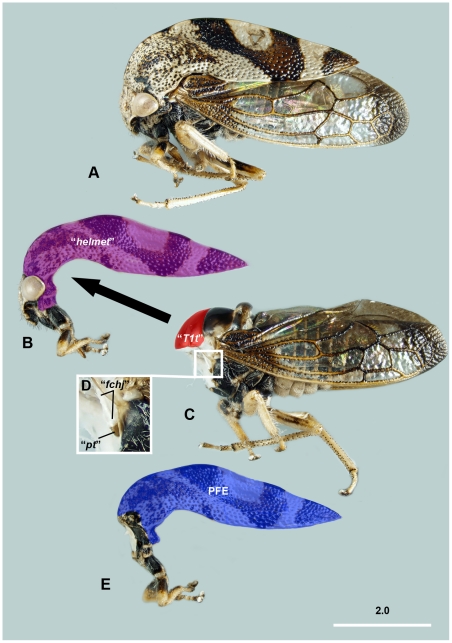
Brightfield images of *Cyrtolobus vau* (Membracidae) showing the body in different stages of subsequent separation of different body parts. A: Habitus, lateral view. B: Fore leg + head + “*helmet*” complex, lateral view, “*helmet*” is annotated with overlay. C: Posterior body parts after removal of fore leg + head + ”*helmet*” complex, lateral view, “*T1 tergum*” is annotated by overlay. D: Anterior margin of T2 tergum and T2 pectus with the “*flexible cuticle of helmet joint*” and the “*pteralium*”. E: T1, lateral view, PFE is annotated by overlay. Abbreviations: “*T1t*” – “*T1 tergum*” ( = median area of T2 tergum); “*fchj*”  = “*flexible cuticle of helmet joint*” ( = intersegmental membrane between T1 and T2); “*pt*” = “*pteralium*” ( = prepectus); PFE = posterior flattened evagination of the pronotum.

Evidence for a spectacular evolutionary novelty was recently reported [1], suggesting that the dorsal prothoracic ornamentation found in treehoppers (Hemiptera: Membracidae)—the so-called “*helmet*” (“***helmet***”: Fig. 1B)—is derived from the (re-)expression of genetic processes responsible for wing development, resulting in the presence of a true articulated (moveable) dorsal appendage on the 1st thoracic segment (T1; prothorax). In extant insects, T1 never bears wing-like structures, and an excited discussion of the implications for this developmental trajectory soon reverberated throughout scientific community (e.g., [2]–[4]).

The hypothesis further stated that this articulated appendage is distinct from the thoracic expansions that evolved in other insect lineages (e.g., horn-like structures in some beetles or other hemipterans), which are non-articulated (immovable) cuticular evaginations. The key feature used by the authors [1] to discriminate between a simple outgrowth and a true appendage was the presence of a jointed articulation—i.e., the well-sclerotized appendage was connected via a band of less sclerotized cuticle (conjunctiva) to a well-sclerotized body region(s), making the appendage movable relative to the rest of the body. The authors [1] described the presence of such a jointed articulation between the “*helmet*” and the dorsal sclerite of the T1, the “*T1 tergum*” (“***T1t***”: Fig. 1C), where the two sclerites are connected via conjunctiva, the “*flexible cuticle of helmet joint*” (“***fchj*** **”**: Fig. 1D).

**Figure 2:**
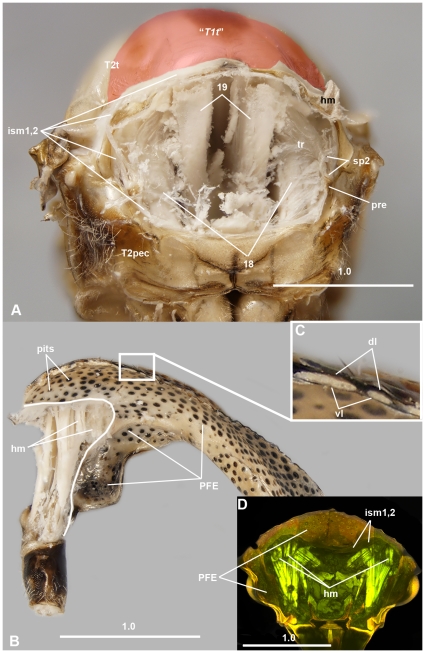
Brightfield images and CLSM micrograph of *Cyrtolobus vau* (Membracidae) showing the articulation between T1 and T2. A: T2, anterior view, “*T1 tergum*” is annotated by overlay. B: T1, median view, white line marks the site of origin of the T1–T2 intersegmental membrane that separates the “*helmet*” for the bi-layered PFE and an anterior, mono-layered area. C: Dorsal and ventral layers of PFE with columnar structures corresponding to external pits. D: T1 and head, posterior view, the posterior part of PFE is removed. Abbreviations: 18 = 1st mesopleuro-mesonotal muscle; 19 = 1st phragmo-2nd phragmal muscle; dl = dorsal layer of PFE; hm = helmet muscle; ism1,2 = intersegmental membrane between T1 and T2; PFE = posterior flattened evagination of the pronotum; pre = prepectus; sp2 = 1st thoracic spiracle; “*T1t*” = “*T1 tergum*” = median area of T2 tergum; T2pec = T2 pectus; tr = trachea; vl = ventral layer of PFE.

**Figure 3:**
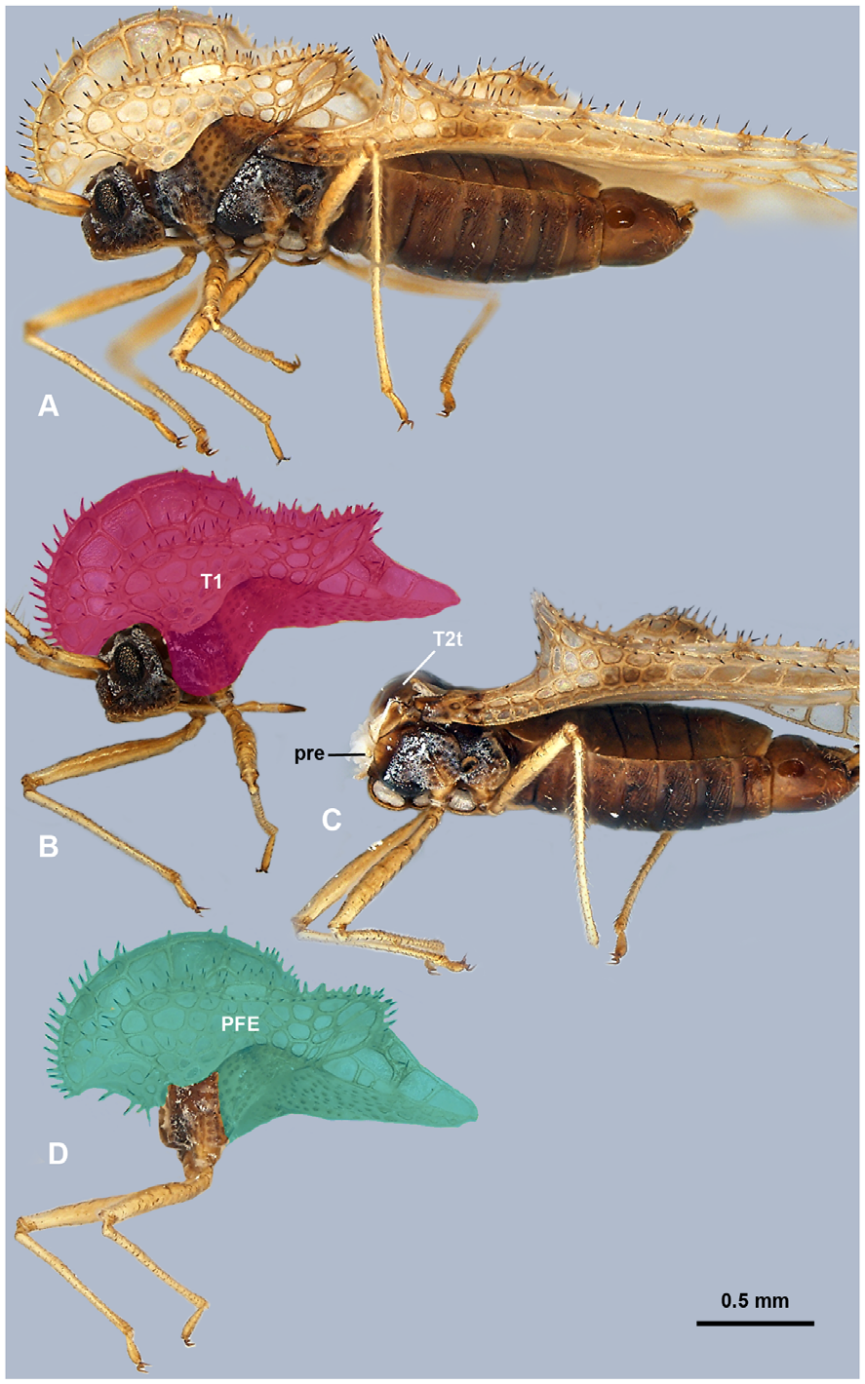
Brightfield images of *Corythucha pallida* (Tingidae) showing the similarity between the membracid and tingid body plan (compare with Figure 1A–E). A: Habitus, lateral view. B: fore leg + head + T1 complex, lateral view, T1 is annotated with overlay. C: Posterior body parts after removal of fore leg + head + T1 complex, lateral view. D: T1, lateral view, PFE is annotated by overlay. Abbreviations: PFE = Posterior flattened evagination of the pronotum; pre = prepectus; T1 = 1st thoracic segment; T2t = T2 tergum.

Based on gene expression and morphological evidence, the T1 dorsal appendage, i.e., the “*helmet*”, was interpreted to be a wing homolog. The authors [1] demonstrated that *nubbin*—a limb developmental gene that facilitates discrimination between wing and other appendage precursors [5], [6]—was expressed in the developing “*helmet*”, as well as in the developing wings of the mesothorax (T2) and metathorax (T3). The morphological evidence supporting the “*helmet*”-wing homology hypothesis focused primarily on the following observations: 1) the “*flexible cuticle of the helmet joint*”, includes a small, embedded, sclerite resembling a pteralium (one sclerite of a cluster that forms a typical wing hinge) that is located at the base of the T2 and T3 wings (**“** ***pt*** **”**: Fig. 1D); 2) the “*helmet*” consists of two layers that are connected via cuticular columns (Fig. 2C); 3) the “*helmet*” has bilateral origin.

We examined the published evidence and determined that neither the textual description nor the associated media in [1] allowed for the reconstruction of the authors' presented morphological observations.

Based on the two non-histological images (see fig. 1e and fig. S2b of [1]), the “*T1 tergum*” is equivalent to the median area of T2 tergum (dorsal plate of T2) of other insects, which corresponds to the site of origin of indirect flight muscles. If the original authors [1] misinterpreted the T2 tergum as the “*T1 tergum*”, then most probably they misinterpreted the real T1 tergum as the “*helmet*” and the T1–T2 articulation as “*flexible cuticle of helmet joint*”. The “*helmet*” would therefore not represent an articulated appendage, but rather would be the equivalent of the T1 tergum or the entire T1 if the T1 tergum is fused with other sclerites of T1 (e.g., if the T1 tergum is fused with the T1 pleura and T1 sternum).

The original authors annotated one paired muscle in their manuscript connecting the “*helmet*” to the “*body*” (see fig. 2f in [1]). The annotated muscle bands clearly insert from the ventral side of the “*helmet*” and arise from the “*T1 tergite*”, according to the annotated image and the description. If we accept that the “*helmet*” is a T1 wing homolog, then it follows that this muscle inserts on the blade of the wing. There is no muscle that inserts on the blades of T2 and T3 wings of any pterygote insect, and so there are two possible explanations for the presence of a tergum-wing blade muscle: 1) the helmet muscle is unique for treehoppers and might develop as a subdivision of a thoracic muscle that is present in other insects, or 2) the “*helmet*” is actually the T1 tergum (or the entire T1), and the muscle is one that normally extends between the T1 tergum and T2 tergum.

The presence of the T1 wing in treehoppers is discussed as an evolutionary novelty that appeared very early during the evolution of Membracidae [1]. Although non-articulated T1 cuticular outgrowths, which resemble wings of T2 and T3 structurally, are present in numerous non-membracid hemipterans (e.g., in Tingidae, Figs. 3A–D), a detailed morphological examination of the Heteroptera pronotum has never been published. Since these cuticular outgrowths were considered as possible precursors of the treehoppers' “*helmet*” [1] a detailed examination of the Heteroptera pronotum is critical for accurate interpretation and contextualization of the results. Here we provide a detailed description of the adult membracid and heteropteran anterior thoracic region using brightfield microscopy, confocal laser scanning microscopy (CLSM), and micro-computed tomography (μ-CT) in order to address outstanding questions about the identity of certain anatomical entities.

## Materials and Methods

Specimens used for dissections and CLSM were collected in North Carolina and Arizona (Table S1) and preserved in 70–80% ethanol. Dissected specimens are deposited in the North Carolina State University Insect Museum; specimens used for μ-CT imaging are deposited in the Zoological Museum Hamburg (Table S1).

Resulting anatomical phenotype descriptions were based on observations made during dissections under stereo (Olympus SZX16 with SDFPL APO 2× PF objective, 230×) and compound (Olympus BX51 with LMPLFLN50× objective; 500×) microscopes. Super Personna razor blades (American Safety Razor Company, Cedar Knolls, NJ, USA) and insect pins were used for dissections. Some specimens were dissected in glycerin, others were critical point dried and dissected on Blu-Tack (Bostik Findley, Wauwatosa, WI, USA).

Confocal laser scanning microscopy (CLSM) and micro-computed tomography (μ-CT) was used to image anatomical structures. Specimens used for CLSM were taken from glycerine, rinsed in 75% alcohol and transferred to type VII, low melting point agarose between 1.5 mm thick, 24×50 mm cover glasses. Specimens where examined with Leica LSM 710 Laser Scanning Confocal Microscope using 488 nm laser for excitation of sample. We collected the autofluorescence of chitin between 405 and 480 nm with two channels using 10× and 20× Plan Achromat objectives.

For μ-CT, the specimen was dehydrated in an ethanol series, critical point dried (Balzers Critical Point Dryer) and mounted with superglue on a metal rod. μ-CT scans were performed at the German Electron Synchrotron Facility (DESY) in Hamburg using a Phoenix nanotom (35 keV, 280 mA). The resulting image stack has a voxel size of 4.05 µm. Three-dimensional reconstructions of the prothoracic anatomy were carried out using Visage Imaging™ Amira® 5.3 software. All discrete structures (sclerites and muscles) were segmented, and individual surface objects were generated based on these segmentations. Surface renderings were done in Autodesk® Maya® 2011 software. The interactive three-dimensional PDF (Fig. S1) file was created using Adobe™ Acrobat® 9 Pro Extended software on basis of the surfaces modified in Maya software.

Anatomical terms in the Results, Table 1, and Table S3 are all mapped to anatomical concepts in a source insect anatomy ontology (Table S2). Although there is currently no Hemiptera-specific anatomy ontology, most anatomical classes of the present description are shared across Pterygota, and hence, we can use an appropriately detailed insect ontology available through the OBO Foundry (http://obofoundry.org). We selected the Hymenoptera Anatomy Ontology [7], because it is presently the only available insect ontology with unambiguous definitions for a majority of the anatomical features (Table S2). *Genus differentia* definitions are proposed for anatomical concepts not currently in any OBO Foundry ontology, following ontology building best practices (e.g., [7]). Throughout this manuscript terms in italics and quotes represent anatomical labels used in the original treehopper T1 wing hypothesis paper [1].

**Table 1:** Interpretation of anatomical structures in the membracid thorax by Prud'homme et al. [1] and by the present paper.

Prud'homme et al. 2011	Present paper
“*flexible cuticle of helmet joint*”	intersegmental membrane between T1 and T2
“*helmet*”	T1 excluding fore legs
“*pteralium*”	prepectus
“*T1 tergite*”	median lobe of T2 tergite
“*helmet muscle*”	pronoto-prophragmal muscle

## Results

### Membracid anterior adult thorax - sclerites and conjunctiva

Sclerites are areas of the insect integument that are well sclerotized; sclerites are rigid plates usually moved relative to each other through the action of muscles. Conjunctivae are areas of the insect integument that are weakly sclerotized; conjunctivae are flexible and hence allow movable contact between sclerites. Labels in bold correspond to image annotations.

**Figure 4:**
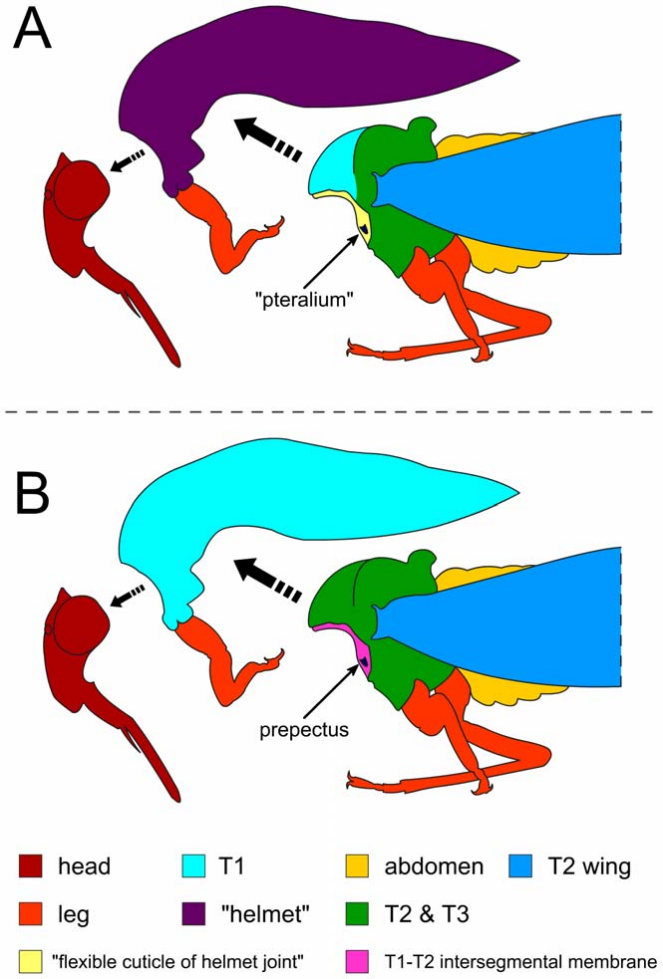
Diagrammatic representation of the major body regions of a treehopper. A: Interpreted by Prud'homme et al. [1]. B: Interpreted in the present study. Tagmata are separated (dashed arrows) to more clearly show anatomical structures.

**Figure 5:**
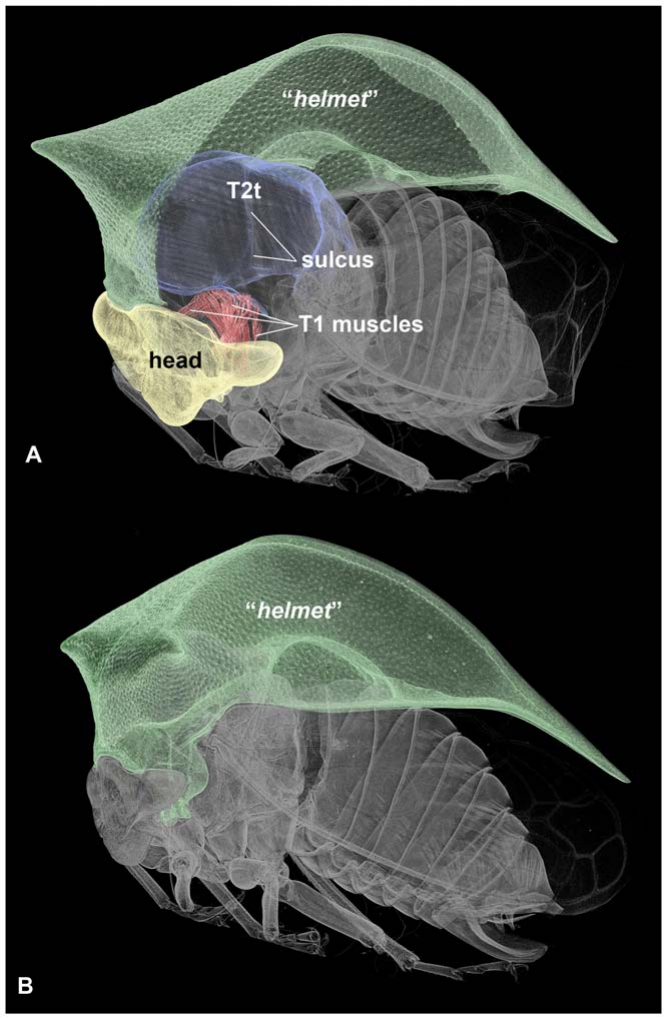
Micro-computed tomography of *Stictocephala bisonia* (Membracidae), showing the relationship between the “*helmet*” and other anatomical structures (volume renderings of μ-CT data). A: Habitus, anterolateral view, left half of “*helmet*” removed. Head, T1 muscles, T2 tergum and “*helmet*” are annotated by overlays. B: Habitus, lateral view, “*helmet*” is annotated by overlay. Abbreviation: T2t = T2 tergum.

**Figure 6:**
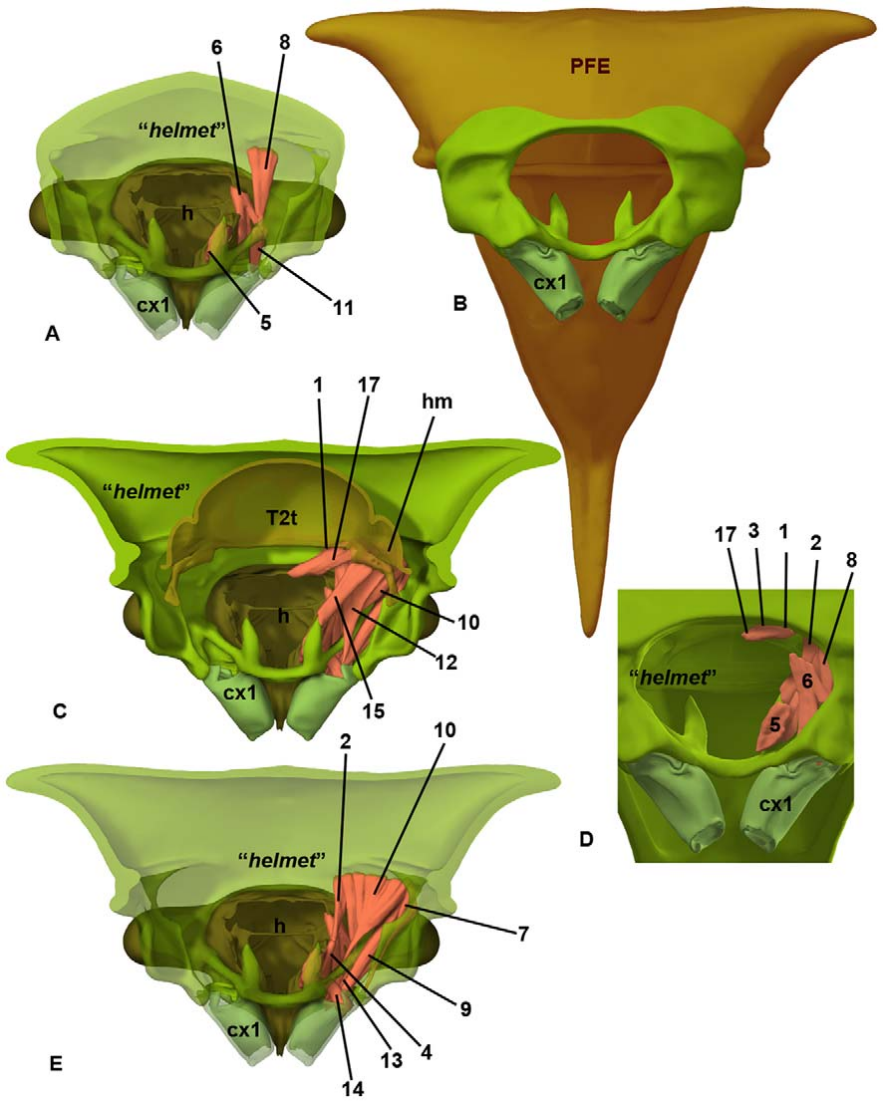
Micro-computed tomography of *Stictocephala bisonia* (Membracidae) showing the relationships between skeletal structures and muscles in T1 (surface rendering of 3D-reconstrution). A: Fore leg + head + “*helmet*” complex, posterior view, PFE in large part removed. B: T1, anterior view, PFE is annotated with overlay. C: Fore leg + head + “*helmet*” complex with T2 tergum, posterior view, “*helmet*” is PFE is partly removed. D: Detail of T1, anterior view. E: Fore leg + head + “*helmet*” complex, posterior view, posterior “*helmet*” is partly removed. Abbreviations: Numbers refer to muscles listed in Table S3; cx1 = procoxa; h = head; hm = helmet muscle; PFE = posterior flattened evagination of the pronotum; T2t = T2 tergum.

**Figure 7:**
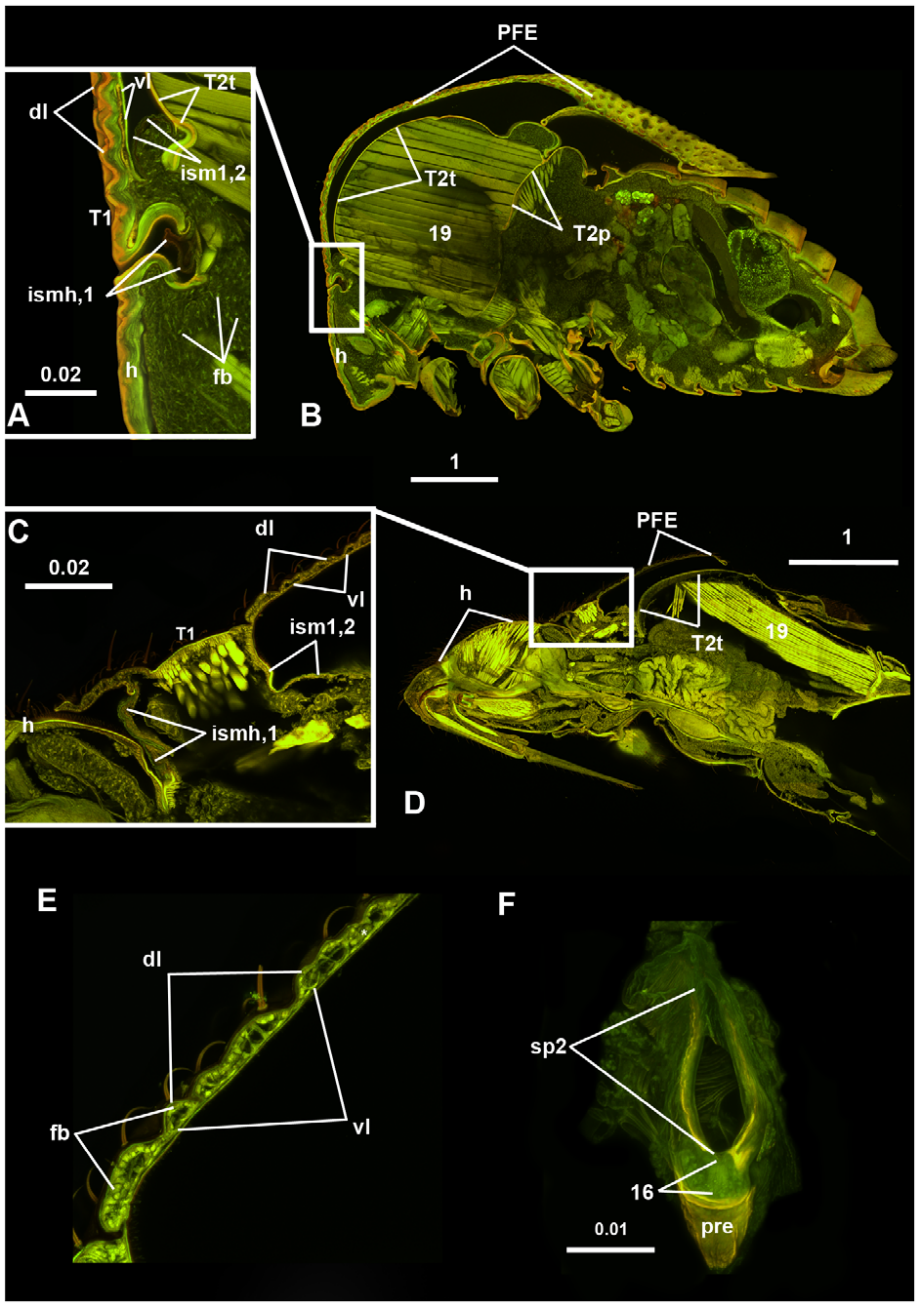
Confocal laser scanning micrographs of hemipteran insects showing the articulation between the “*helmet*”, head and T2 tergum and the structure of PFE. A, B: *Ceresa* sp. (Membracidae) A: Head-“*helmet*” and “*helmet*”-T2 tergum joints, sagittal section, anterior left, detail of Fig. 6B. B: Body, sagittal section, anterior to the left. C–E: *Leptocoris trivittatus* (Coreidae). C: Head-“*helmet*” and “*helmet*”-T1 joint, sagittal section, anterior to the left, detail of Fig. 6D; D: Body, sagittal section, anterior to the left. E: PFE, sagittal section; F: *Atymna querci* (Membracidae), 1st thoracic spiracle with *pteralium* ( = prepectus). Abbreviations: 16 = Occlusor muscle of 1st thoracic spiracle; 19 = 1st mesopleuro-mesonotal muscle; dl = dorsal layer; fb = fat body cell-like structures; h = head; ism1,2 = intersegmental membrane between T1 and T2; ismh,1 = intersegmental membrane between head and T1; PFE = posterior flattened evagination of T1; pre = prepectus; sp2 = 1st thoracic spiracle; T1 = 1st thoracic segment; T2p = T2 postnotum; T2t = T2 tergum; vl = ventral layer.

The “*helmet*”, together with the fore legs and the head, compose an anterior body complex that is moveably attached to the rest of the body via a conjunctiva (Figs. 1A, B, 4A, 5A, B, 6A, C–E, S1), in this case the “*flexible cuticle of the helmet joint*” (Fig. 4B; **fchj**: Fig. 1D; **ism1,2**: Figs. 2A, D, 7A). The conjunctiva extends along the anterior margin of the body except the head + fore leg + “*helmet*” complex and separates a narrower anterior and a larger posterior area on the “*helmet*” (Figs. 2B, 6B, S1).

**Figure 8:**
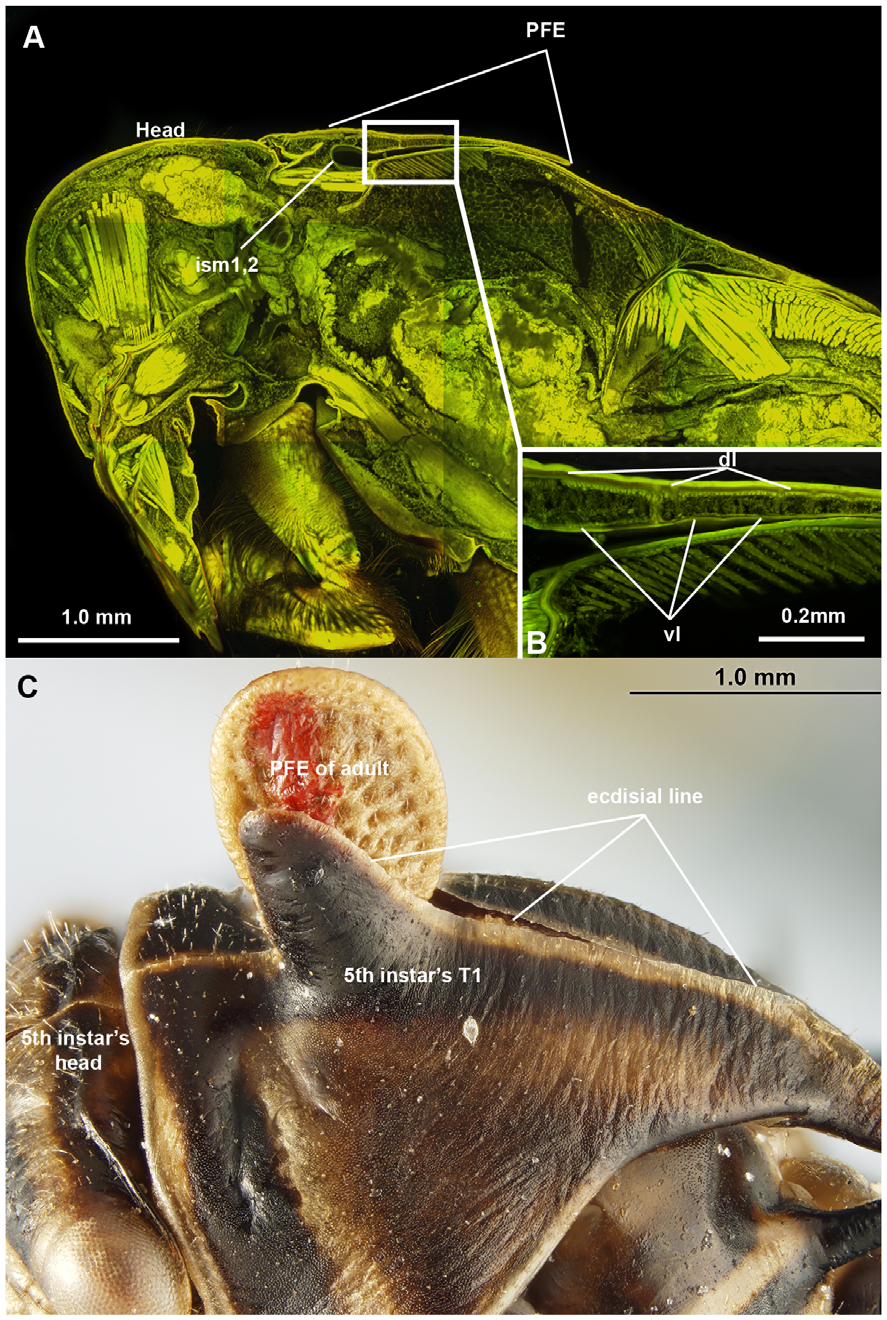
Confocal laser scanning micrographs and brightfield image of hemipteran insects showing the separated layers of PFE in pharate adult and the ecdysial line of 5th instar. A: Notonectidae sp., (Notonectidae) body, sagittal section, anterior to the left. B: PFE, sagittal section, anterior to the left. C: *Platycotis vittata* (Membracidae), head and T1 of 5th instar during ecdysis (please note the presence of red mark on the PFE of emerging adult). Abbreviations: ism1,2 = intersegmental membrane between T1 and T2; PFE = posterior flattened evagination of the pronotum; vl = ventral layer of PFE; dl = dorsal layer of PFE.

**Figure 9:**
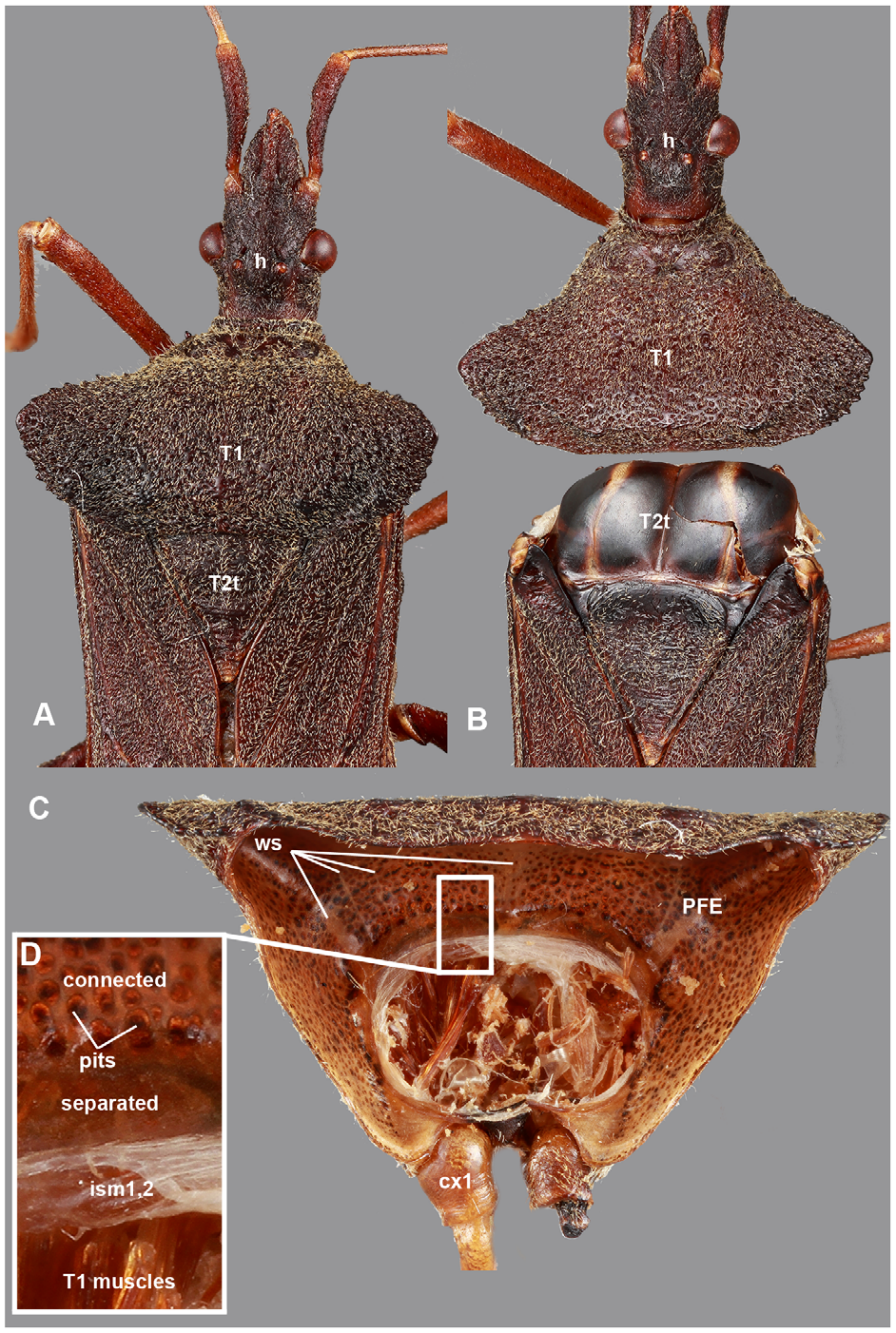
Brightfield images of *Leptoglossus fulvicornis* (Coreidae) showing the relationship and articulation between T1 and T2. A: Anterior half of body, dorsal view, anterior to the top. B: Anterior half of body, dorsal view, anterior to the top, fore leg + head + T1 complex detached from the posterior part of body. C: T1, posterior view. D: anterior margin of PFE, posterior view, detail of Fig. 7C. Abbreviations: cx1 = procoxa; PFE = posterior flattened evagination of the pronotum; T1 = 1st thoracic segment; T2t = T2 tergum; h = head; ism1,2 = intersegmental membrane between T1 and T2; ws = wing vein-like structure.

The anterior, narrower area of the “*helmet*” is mono-layered, whereas the posterior, more extended area is a bilayered, posterior, flattened, cuticular evagination (PFE) (**PFE**: Figs. 1E, 2B, 6B, 7B). The dorsal (external) and ventral (internal) layers of the PFE are entirely separated in freshly emerged adults (e.g., **dl**, **vl**: Fig. 8B), but separated only extremely anteriorly (e.g., **separated**: Fig. 9D) and connected posteriorly (e.g., **connected**: Fig. 9D) with columnar structures in older adults (**dl**, **vl**: Figs. 2C, 7A). The columnar structures correspond to distinct pits both on the internal (ventral) and external (dorsal) surfaces of the PFE (**pits**: Fig. 2B). The lumen between the layers is continuous with the cavity surrounded by the helmet and hence is filled with similar, fat body cell-like structures that are found between the thoracic muscles (**fb**: Fig. 7A). In mature adults, hollow, trachea-containing, elongate structures extend along the PFE.

The “*helmet*” is articulated via conjunctivae and articular surfaces (areas that are located on a sclerite and that make movable, direct contact with another sclerite) with the fore leg and the head (**ismh,1**: 7A). As described above, the “*helmet*” is connected to the posterior body by the “*flexible cuticle of the helmet joint*” (Fig. 4B; **fchj**: Fig. 1B; **ism1,2**: Figs. 2A, D, 7A). The dorsal part of the conjunctiva extends along the anterior margin of a horizontal, slightly convex sclerite, the T2 tergum (**T2t**: Figs. 2A, 5A, 6C; **mesonotum**: Fig. S1) whereas the ventral part extends along the anterior margin of a U-shaped sclerite, the T2 pectus (**T2pec**: Fig. 2A). “*T1 tergum*” (“***T1t***”: Figs. 1C, 2A) is set of by two sulci (**sulcus**: Fig. 5A) from the posterolateral part of T2 tergum (**mesonotum**: Fig. S1), but otherwise it is continuous with the posterior part of T2 tergum.

The “*pteralia*” (Fig. 4A; **“** ***pt*** **”**: Fig. 1D; **pre**: Figs. 2A, 7F) are small sclerites that are situated laterally on the “*flexible cuticle of helmet joint*” just ventrally of the 1st thoracic spiracles (the anterior-most opening of the respiratory system) (**sp2**: Figs. 2A, 7F).

### Membracid anterior adult thorax - muscles

16 muscles attach to the “*helmet*” (Fig. S1). All of these muscles arise anterior to the “*flexible cuticle of helmet joint*” (Figs. 2B, D, 6A, E, S1), from the mono-layered area. One of these is the “*helmet muscle*” (**hm**: Figs. 2A, B, D, 6C; Fig. S1), which connects the “*helmet*” to the T2 tergite. Among the remaining 15 muscles (**1–15**: Figs. 6A, C–E; **m1–m15**: Fig. S1), two connect the helmet to the anterior margin of T2 tergum (muscles 1, 15), five with the head (muscles 2–6), and eight with the fore leg (muscles 7–14). The “*pteralium*” is connected by a muscle (muscle 16) to the 1st thoracic spiracle (**16**: Fig. 7F). Among the nine muscles attaching to the T2 tergum the three largest are muscles 18, 19 and 20 (**18**, **19**: Fig. 2A). Muscles 18 and 19 arise from “*T1 tergum*” (**“T1t”**: Fig. 2A), whereas muscle 20 arises just laterally of the sulci defining “*T1 tergum*” (**sulcus**: Fig. 5A).

### Reconciliation of anatomical concepts

Our reconciliation of anatomical concepts used by the original authors [1] is provided in Table 1 and Fig. 4. Based on the articulating sclerites and muscles, the “*helmet*” is actually the entire T1, excluding the fore legs (**prothorax incl. helmet**: Fig. S1).

Muscles 18 and 19 comprise the dorsoventral and longitudinal indirect flight muscles of T2. The line extending along the border of the site of origin of the muscles separates “*T1 tergum*” (where muscle 19 originates) and the two lateral areas of T2 tergum (where the paired muscle 18 originates). “*T1 tergum*” is therefore equivalent with the anteromedian area of T2 tergum and is not part of T1.

The “*pteralium*” serves as the site of origin of the occlusor muscle of the 1st thoracic spiracle (muscle 16; when the muscle is contracted the spiracle is closed, and when relaxed the spiracle is open). The “*pteralium*” is therefore the prepectus, a sclerite that is located on the intersegmental membrane between T1 and T2 and serves as the site of origin of the occlusor muscle of the 1st thoracic spiracle.

The “*flexible cuticle of the helmet joint*” is actually the intersegmental membrane between T1 and T2.

The “*helmet muscle*” arises from the T2 tergum anteroventrally of the intersegmental membrane between T1 and T2. The area on T2 tergum that extends anteriorly (ventrally) of the intersegmental membrane is the prophragma. Therefore the “*helmet muscle*” is actually the pronoto-prophragmal muscle, which connects the T2 tergum with the real T1 tergum.

### T1 of other adult hemipterans

The T1 of non-membracid hemipterans shares numerous characteristics with the T1 of membracids: 1) the PFE is present in all examined hemipterans and is delimited anteriorly by the intersegmental membrane between T1 and T2 (**ism1,2**: Figs. 7C, 9D). The length of PFE is variable in Hemipterans (it is less developed in non-membracid Auchenorrhyncha and more developed in Heteroptera (**PFE**: Figs. 3D, 7D, 9C)); 2) the two layers of PFE are separated along the entire length in freshly emerged adults (Fig. 9A), whereas in older adults the layers remain separated only along an anterior narrow area (**separated**: Fig. 9D) and are connected with columnar structures posteriorly (**connected**: Fig. 9D) that correspond to pits (**pits**: Fig. 9D); 3) fat body cell-like cellular structures are located between the two layers (**fb**: Fig. 7E); 4) trachea- and nerve-containing hollow, longitudinal, wing vein-like structures that typically extend along the length of the PFE in adults (**ws**: Fig. 9C).

### T1 of immature hemipterans

In hemipteran instars, the PFE is either absent or the layers are separated from one another. In all nymphal stages a median ecdysial line is present dorsally on the thoracic segments and the head. During ecdysis the old cuticle breaks open along this line (Fig. 8C).

Wing buds (precursors of wings) on T2 and T3 are present only on 3rd–5th instars and are absent in the 1st and 2nd instars. There is no paired structure present in T1 of the 1st and 2nd instar.

## Discussion

Based on these results, we conclude that there is no articulated dorsal appendage on the T1 of treehoppers. The putative prothoracic wing articulation described by the original authors [1] is actually the intersegmental membrane between the pro- and mesothorax. This conjunctiva provides the “*helmet*” the mobility that typically exists between sclerites and would give the impression that an appendage articulation could be involved (e.g. the movie file (Supplementary Movie 1) published in [1]. Furthermore, the position of the proposed “*pteralia*” next to the tracheal opening (spiracle) is consistent with this structure instead being the prepectus, a sclerite that serves as the attachment site for the occlusor muscle that closes the spiracle.

### What is the “helmet”?

Based on the comparative morphology of sclerites and the position of thoracic muscles, we conclude that the “*helmet*” is equivalent to the entire T1, excluding the fore legs. Thus any observations and hypotheses made on “*helmet*” development must refer more broadly to the development of T1 itself. A recent opinion [8], made available online early while we revised our manuscript, independently corroborates our conclusions based on the author's observations of *Publilia modesta* (the species examined in [1]) and a relatively closely related taxon (Hemiptera: Cicadellidae: *Pagaronia* sp.). The bilayered, wing-like, posterior evagination, or PFE, of the “*helmet*” is equivalent to the cuticular evaginations observed in other insects, e.g., horns in certain beetle species [9]. The external resemblance of this evagination to a veined wing in some treehoppers is a result of a wing vein-like tracheal and hemolymph nutrient support system [10].

### Is the PFE an evolutionary novelty?

The presence of the PFE is widely accepted as a membracid characteristic (Deitz and Wallace, Treehoppers website; http://purl.oclc.org/NET/treehoppers/index), one that was either in the ground plan of the family and lost twice, or evolved independently in two separate lineages (the former scenario being considered more likely [11]). Although there is no articulated appendage on the treehopper T1, the PFE clearly shares numerous structural and at least one genetic attribute with T2 and T3 wings: 1) the PFE is flattened, and hence the two layers of PFE are located relatively close to each other; 2) the layers are separated in newly eclosed adults but are connected via columnar structures in older adults; 3) hollow, trachea-containing, longitudinal structures extend along the length of PFE in mature adults; 4) the lumen of the flattened evagination is continuous with the body cavity and hence contains fat body cell-like structures. Most of these structural attributes are, however, present in the PFE of other hemipterans (i.e., this structure is not unique to Membracidae).

The original authors [1] relied on two lines of evidence to support a bilateral origin of the “*helmet*”, which was offered as further evidence of the “*helmet*'s” novel, wing-like nature: the presence of paired wingbud-like structures in the pronotum of the 2nd instar (see figs S3, S4 in [1]) and the presence of the median line on the pronotum in other instars. Our observations of 2nd instar membracids failed to yield bud-like structures, nor were these structures discussed in any of the literature we reviewed (e.g., [12]). We posit that the apparent bud-like structures in [1] might represent a sampling artifact due to the diagonal nature of the section they marked on the specimens. We also acknowledge that the PFE, along with T2 and T3 wing primordia, are absent from 1st and 2nd instars and appear only in the 3rd instar. Therefore, it is likely an extraordinary challenge to study PFE development during the development of 1st and 2nd instars.

The median, longitudinal line on the nymphal pronotum is an ecdysial line [12] (Fig. 8C; observed also in [8]). Structures that span a midline in adult insects are common [10], each serving at least one of an array of functions (e.g., an internal phragma might separate and serve as site of origin for the longitudinal indirect flight muscles in some Hymenoptera, which creates an external line).

We therefore conclude that the treehopper PFE is most likely not bilateral in origin, and that there is no structural difference between the treehopper PFE and PFEs present in other hemipterans. Membracid T1 ornamentation is therefore not different from prothoracic ornamentations present in other hemipterans, e.g., Tingidae (Figs. 3A–D), and the presence of PFE is not an evolutionary novelty for treehoppers.

### PFE: a simple evagination controlled by appendage genes

Prud'homme et al. [1] examined the expression of three genes known to effect wing development in *Drosophila* (*nubbin*, *distal-less*, and *homothorax)* and a gene known to repress wing development (*Sex combs reduced* (*Scr*)) during the development of the treehopper PFE. As their leading evidence the authors argued that *nubbin* is a wing-specific gene, therefore its observed expression in the PFE around the same time as the wing favors it employing wing development pathways. The evolution of *nubbin* expression has been assessed across embryos from multiple arthropod species, the most closely related being the milkweed bug *Oncopeltus fasciatus* [5], [6], [13], [14]. This research determined that *nubbin* is evolutionarily labile, with ancestral involvement in arthropod limb segmentation. In *O. fasciatus* it is expressed in the central nervous system and both leg and head appendages. Although focus on this gene in *Drosophila* has been its expression in the wing and central nervous system, it also is involved in early leg development [15], [16]. Therefore, like *Distal-less* and *homothorax* that are major genes involved in the formation of non-flattened T1 evaginations in beetles [17], *nubbin* does not necessarily indicate that the PFE is serially homologous to the wings of T2 and T3, but instead shows the shared genetic regulation between real appendages and simple evaginations. Prud'homme et al. [1] also found that *Scr* is not only expressed in T1 but that the protein is functional. They posit that *Scr* is therefore not involved in allowing the formation of a prothoracic wing and instead propose that it must be a gene downstream of *Scr*. An alternative interpretation is that the treehopper PFE is not derived from wing formation pathways. In contrast, *Scr* could play a role in formation of the PFE independent of its role in wing development, as up-regulation of *Scr* plays an integral role in enhancing beetle T1 horn length during pupal stages [18] and in modifications of T1 shape in *Oncopeltus* [19].

Although their data do not provide convincing support for the PFE being serially homologous to wings, the expression of these genes in combination emphasize the appendage-like nature of the PFE, a simple, non-articulated cuticular evagination, and raise the possibility that developmental pathways similar to those in the wing may have been co-opted.

### Accessibility of morphological data

The call for data standards in descriptive biology, e.g., minimum information checklists for phenotype representation and imaging, has recently become louder [20], [21]. The treehopper pronotal wing hypothesis yields examples of misinterpretation that could have been avoided through updated best practices in phenotype knowledge representation and the broader development of anatomical references. Necessary and sufficient definitions for insect anatomical entities, for example, could have prevented the misinterpretation of the T1, T2 tergum, intersegmental membrane between T1 and T2, and the prepectus. To attach definitions or image annotations for all anatomical entities (including those, that are used in the definitions of structures used in the text), however, is equivalent with attaching an entire anatomy glossary.

The developmental origin for evolutionary novelties like PFEs is intriguing, especially when considering the diversity of gene co-option involved in repeated evolution of similar structures. We hope the morphological observations in this study helps to correctly guide future research on these systems. Broad-scale accessibility of morphology data requires that we pursue new methods in the way we report phenomic observations.

## Supporting Information

Figure S1 **Interactive three-dimensional PDF: Micro-computed tomography of** ***Stictocephala bisonia*** **(Membracidae) showing the relationships between skeletal structures and muscles in T1 (surface rendering of 3D-reconstrution).**
(PDF)

Table S1 **Specimens examined.**
(DOCX)

Table S2 **Anatomical terms used in our description, cross-referenced to an ontological definition.** URI = Uniform Resource Identifier.
(DOCX)

Table S3 **Observed muscles.** URI = Uniform Resource Identifier.
(DOCX)
