# Supplementary figures and images for: On Dorsal Prothoracic Appendages in Treehoppers (Hemiptera: Membracidae) and the Nature of Morphological Evidence
Source: PLoS One. 2012 Jan 17;7(1):e30137. doi: 10.1371/journal.pone.0030137 (PMC3260216; doi:10.1371/journal.pone.0030137)

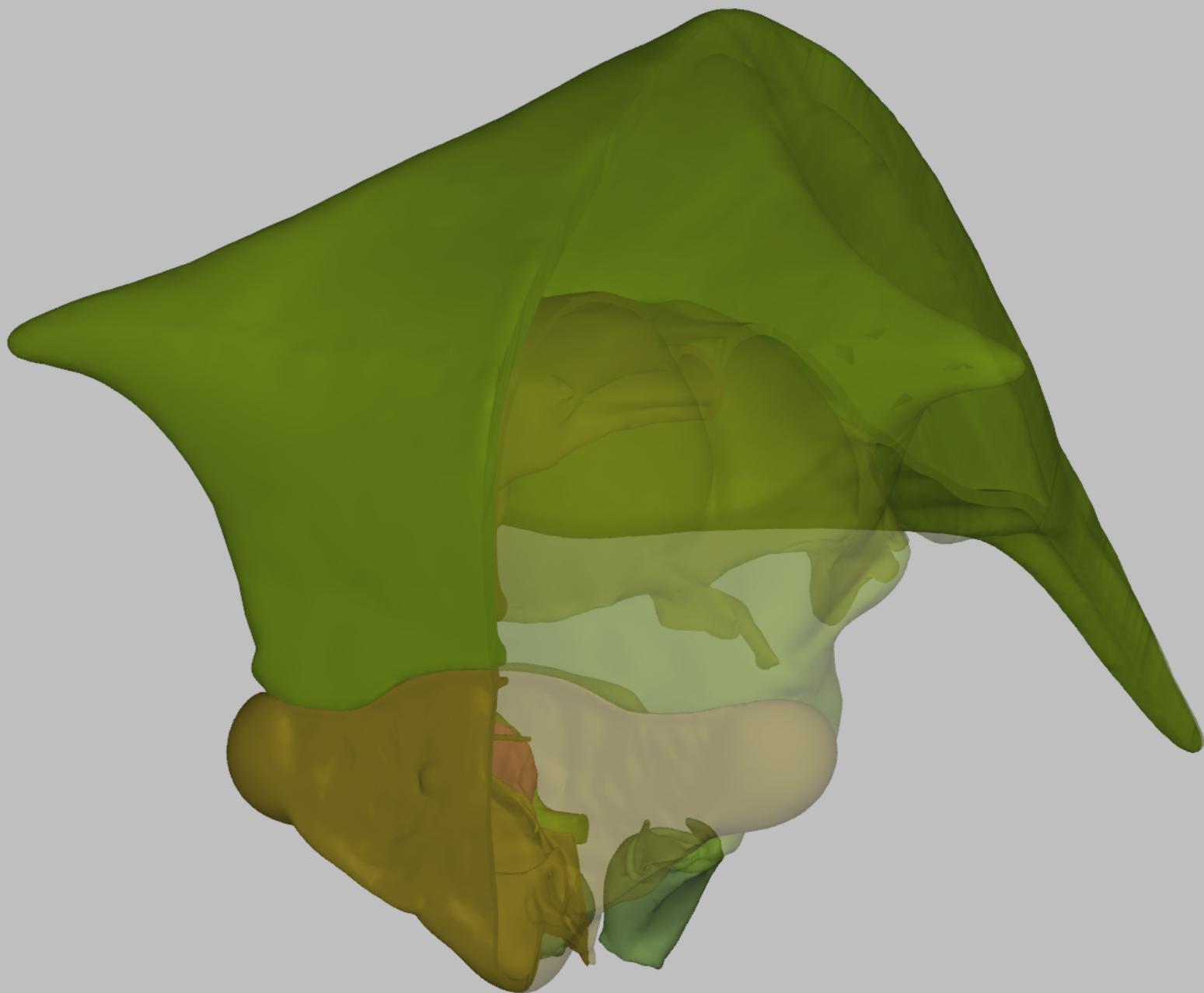

Supplement: Figure S1 — Interactive three-dimensional PDF: Micro-computed tomography of Stictocephala bisonia (Membracidae) showing the relationships between skeletal structures and muscles in T1 (surface rendering of 3D-reconstrution). (PDF) [file pone.0030137.s001.pdf]
