# Supplementary material for: On Dorsal Prothoracic Appendages in Treehoppers (Hemiptera: Membracidae) and the Nature of Morphological Evidence
Source: PLoS One. 2012 Jan 17;7(1):e30137. doi: 10.1371/journal.pone.0030137 (PMC3260216; doi:10.1371/journal.pone.0030137)
Supplement: Table S1 — Specimens examined. (DOCX) [file pone.0030137.s002.docx]

Table S1. Specimens used in this study.

| **Species** | **Identifiers (namespace=NCSU)** | **Methods** |
| --- | --- | --- |
| *Acrosternum hilare* (Pentatomidae) | 55693 | Dissection |
| *Atymna querci* (Membracidae) | 53801, 55688 | Dissection |
| *Ceresa sp.* (Membracidae) | 55678, 55679 | Dissection, CLSM |
| *Corythucha pallida* (Tingidae) | 43250, 43251 | Dissection |
| *Cyrtolobus tuberosus* (Membracidae) | 28911, 28912 | Dissection |
| *Cyrtolobus vau* (Membracidae) | 53800, 53802–53804 | Dissection |
| *Leptocoris trivittatus* (Coreidae) | 55675, 55680 | Dissection, CLSM |
| *Leptoglossus fulvicornis* (Coreidae) | 17998, 17997 | Dissection, CLSM |
| *Lygus lineolaris* (Miridae) | 55681, 55682 | Dissection |
| *Magicicada septemdecim*  (Cicadidae) | 45492, 45491 | Dissection |
| *Neoperkinsiella guaduae* (Delphacinae) | 45460, 45494 | Dissection |
| Notonectidae sp*.* (Notonectidae) | 43286, 43285, 43284 | Dissection, CLSM |
| *Platycotis vittata* (Membracidae) | 55684, 55683 | Dissection, CLSM |
| *Rhagovelia sp.* (Veliidae) | 17979, 17980 | Dissection |
| *Stictocephala bisonia*  (Membracidae) | 45943 | µCT |
| *Xantholobus muticus* (Membracidae) | 55696, 55692 | Dissection |
| *Zanna madagascariensis* (Fulgoridae) | 45493 | Dissection |
