# Supplementary material for: On Dorsal Prothoracic Appendages in Treehoppers (Hemiptera: Membracidae) and the Nature of Morphological Evidence
Source: PLoS One. 2012 Jan 17;7(1):e30137. doi: 10.1371/journal.pone.0030137 (PMC3260216; doi:10.1371/journal.pone.0030137)
Supplement: Table S2 — Anatomical terms used in our description, cross-referenced to an ontological definition. URI = Uniform Resource Identifier. (DOCX) [file pone.0030137.s003.docx]

Table S2. Anatomical classes.

| **label** | **concept** | **URI or concept ID + ontology URL** |
| --- | --- | --- |
| anterior thoracic spiracle | The spiracle that is located on the border of the pronotum and mesopleuron. | <http://purl.obolibrary.org/obo/HAO_0000582> |
| area | The anatomical structure that is delimited by material or immaterial anatomical entities. | <http://purl.obolibrary.org/obo/HAO_0000146> |
| body | The anatomical cluster that is composed of the whole organism but which excludes the antennae, legs and wings. | <http://purl.obolibrary.org/obo/HAO_0000182> |
| cavity | Non-material anatomical entity of three dimensions, that is generated by morphogenetic or other physiologic processes; is surrounded by one or more anatomical structures; contains one or more organism substances or anatomical structures. | <http://purl.obolibrary.org/obo/CARO_0000005> |
| conjunctiva | The area of the integument that is weakly sclerotized, with thin exocuticle. | <http://purl.obolibrary.org/obo/HAO_0000221> |
| cuticle | The acellular anatomical structure that is the external layer of the integument (covers the entire body surface as well as lines ectodermal invaginations such as the stomodeum, proctodeum and tracheae) and produced by the epidermal cells. | <http://purl.obolibrary.org/obo/HAO_0000240> |
| cuticular evagination | The area that is an outpouching of the integument. | <http://purl.obolibrary.org/obo/HAO_0001924> |
| fat body cell | A cell whose primary function is intermediary metabolism. | <http://purl.obolibrary.org/obo/CL_0000565> |
| 1st thoracic spiracle | The spiracle that is located on the border of the pronotum and mesopleuron. | <http://purl.obolibrary.org/obo/HAO_0000582> |
| “*flexible cuticle of the helmet joint*" | The intersegmental membrane that is located between the mesopectus and the pronotum. | <http://purl.obolibrary.org/obo/HAO_0001791> |
| fore leg | The leg that is located on the prothorax. | <http://purl.obolibrary.org/obo/HAO_0000349> |
| head | The tagma that is located anterior to the thorax. | <http://purl.obolibrary.org/obo/HAO_0000397> |
| “*helmet*" | The thoracic segment that is located between the head and the mesothorax is composed of the pronotum, the propectus. | <http://purl.obolibrary.org/obo/HAO_0000874> excluding <http://purl.obolibrary.org/obo/HAO_0000349> |
| integument | The anatomical system that forms the covering layer of the animal, ectodermal in origin and composed of epidermal cells producing the cuticle. | <http://purl.obolibrary.org/obo/HAO_0000421> |
| intersegmental membrane | The conjunctiva that connects two segments. | <http://purl.obolibrary.org/obo/HAO_0000434> |
| intersegmental membrane between T1 and T2 | The intersegmental membrane that is located between the mesopectus and the pronotum. | <http://purl.obolibrary.org/obo/HAO_0001791> |
| leg | The anatomical cluster that is composed of the coxa and all distal leg segments and is connected to the pectus. | <http://purl.obolibrary.org/obo/HAO_0000494> |
| line | The anatomical structure that is linear. | <http://purl.obolibrary.org/obo/HAO_0001586> |
| lumen | Non-material anatomical entity of three dimensions, that is generated by morphogenetic or other physiologic processes; is surrounded by one or more anatomical structures; contains one or more organism substances or anatomical structures. | <http://purl.obolibrary.org/obo/CARO_0000005> |
| margin | The line that delimits the periphery of an area. | <http://purl.obolibrary.org/obo/HAO_0000510> |
| muscle | Portion of tissue composed of contractile fibers. | <http://purl.obolibrary.org/obo/ZFA_0005145> |
| pits | The patch that is impressed and corresponds to an apophysis. | <http://purl.obolibrary.org/obo/HAO_0000718> |
| plates | The area of the integument where the cuticle is well sclerotized with thick exocuticle. | <http://purl.obolibrary.org/obo/HAO_0000909> |
| prepectus | The intersegmentalia that is located on the mesopectus-pronotum intersegmental membrane and serves as the site of origin of the anterior thoracic spiracle occlusor muscle. | <http://purl.obolibrary.org/obo/HAO_0000811> |
| prophragma | The phragma that extends along the anterior margin of the mesonotum. | <http://purl.obolibrary.org/obo/HAO_0000855> |
| “*pteralia*", "*pteralium*" | The intersegmentalia that is located on the mesopectus-pronotum intersegmental membrane and serves as the site of origin of the anterior thoracic spiracle occlusor muscle. | <http://purl.obolibrary.org/obo/HAO_0000811> |
| sclerite | The area of the integument where the cuticle is well sclerotized with thick exocuticle. | <http://purl.obolibrary.org/obo/HAO_0000909> |
| segment | The anatomical cluster that is connected to other segments via conjunctivae and muscles and is delimited by its sclerites. | <http://purl.obolibrary.org/obo/HAO_0000929> |
| spiracle | The anatomical cluster that is composed of the distal end of the trachea and the margin of the sclerite or conjunctiva surrounding the spiracular opening. | <http://purl.obolibrary.org/obo/HAO_0000950> |
| sulcus | The groove that corresponds to a ridge. | <http://purl.obolibrary.org/obo/HAO_0000978> |
| T1 | The thoracic segment that is located between the head and the mesothorax is composed of the pronotum, the propectus and the fore leg. | <http://purl.obolibrary.org/obo/HAO_0000874> |
| “*T1 tergum*" | The area that is located medially on the mesonotum and is delimited laterally by sulci separating the site of origin of the first mesopleuro-mesonotal and the mesono-mesolaterophragmal muscles. | n/a (Auchenorrhyncha or Heteroptera specific structure that is missing from the available source ontologies) |
| T2 | The thoracic segment that is located between the prothorax and the metathorax and is delimited by the mesopectus and the mesonotum. | <http://purl.obolibrary.org/obo/HAO_0000583> |
| T2 tergum | The area that is limited anteriorly by the pronotum, laterally by the basalare, axillary sclerites, subalare and the mesopectus and posterolaterally by the mesopostnotum and the metanotum. | <http://purl.obolibrary.org/obo/HAO_0000556> |
| T2 wing | The wing that is located on the mesothorax. | <http://purl.obolibrary.org/obo/HAO_0000351> |
| T3 wing | The wing that is located on the metathorax. | <http://purl.obolibrary.org/obo/HAO_0000400> |
| tergite | The sclerite that is located on the tergum. | <http://purl.obolibrary.org/obo/HAO_0001005> |
| tergum | The area that is located on the integument and is dorsal of the ventral diaphragm. | <http://purl.obolibrary.org/obo/HAO_0001006> |
| thoracic segment | The body segment that is located in the thorax. | <http://purl.obolibrary.org/obo/HAO_0001013> |
| thorax | The tagma that is composed of the prothorax, mesothorax and metathorax and is located between the head and the abdomen. | <http://purl.obolibrary.org/obo/HAO_0001015> |
| vein | The area that is linear and sclerotized and acts as support for the wing membrane. | <http://purl.obolibrary.org/obo/HAO_0001095> |
| wing | The area that is located in between the notum and the pleuron and is composed of the wing base and wing blade. | <http://purl.obolibrary.org/obo/HAO_0001089> |
| wing bud | Region of dorsal mesothoracic disc that will develop into the wing blade. | FBbt:00006029; <http://obo.cvs.sourceforge.net/viewvc/obo/obo/ontology/anatomy/gross_anatomy/animal_gross_anatomy/fly/fly_anatomy_XP.obo> |
| wing vein | The area that is linear and sclerotized and acts as support for the wing membrane. | <http://purl.obolibrary.org/obo/HAO_0001095> |
| wing | The area that is located in between the notum and the pleuron and is composed of the wing base and wing blade. | <http://purl.obolibrary.org/obo/HAO_0001089> |

**^2^** URIs resolve to definitions and included metadata from the supporting anatomy ontologies.
