# Supplementary material for: On Dorsal Prothoracic Appendages in Treehoppers (Hemiptera: Membracidae) and the Nature of Morphological Evidence
Source: PLoS One. 2012 Jan 17;7(1):e30137. doi: 10.1371/journal.pone.0030137 (PMC3260216; doi:10.1371/journal.pone.0030137)
Supplement: Table S3 — Observed muscles. URI = Uniform Resource Identifier. (DOCX) [file pone.0030137.s004.docx]

Table S3. Muscles.

|  | | | | **Membracid specific muscle insertions** | |
| --- | --- | --- | --- | --- | --- |
| **Label used in present description** | **Preferred label in source ontology** | **URI / Definition^1^** | **Figure** | **Origin** | **Insertion** |
| helmet muscle | pronoto-prophragmal muscle | [http://purl.obolibrary.org/obo/HAO_0000850](http://purl.obolibrary.org/obo/HAO_0000850;) | 2A, 2B, 2D, 6C, S1 | posterior margin of pronotum | lateral prophragma |
| muscle 1 |  | The pronotal muscle that arises from the anterior margin of the mesonotum (anteriorly of the prophragma) and inserts anteriorly on the pronotum. | 6C, 6D, S1 |  |  |
| muscle 2 |  | The cranial muscle that arises laterally from anterior margin of pronotum and inserts on the occipital condyle. | 6D, 6E, S1 |  |  |
| muscle 3 | pronoto-postoccipital muscle | <http://purl.obolibrary.org/obo/HAO_0000847> | 6D, S1 | posterior rim of pronotum | postocciput close to midline |
| muscle 4 |  | The cranial muscle that arises medially from profurcal arm and inserts on the occipital condyle. | 6E, S1 |  |  |
| muscle 5 | median profurco-postoccipital muscle | [http://purl.obolibrary.org/obo/HAO_0001892](http://purl.obolibrary.org/obo/HAO_0001892;) | 6A, 6D, S1 | anterior area of dorsal profurcal arm | cranium ventrolateral of occipital foramen |
| muscle 6 | propleuro-postoccipital muscle | [http://purl.obolibrary.org/obo/HAO_0000859](http://purl.obolibrary.org/obo/HAO_0000859;) | 6A, 6D, S1 | dorsal and anterior rim of propleuron | cranium laterally of occipital foramen |
| muscle 7 |  | The procoxal muscle that arises from the pronotum and inserts on the lateral procoxal condyle. | 6E, S1 |  |  |
| muscle 8 | pronoto-procoxal muscle | <http://purl.obolibrary.org/obo/HAO_0000848> | 6A, 6D, S1 | lateral pronotum | anterior coxal rim |
| muscle 9 |  | The protrochanteral muscle that arises from the pronotum and inserts on the depressor tendon of the protrochanter. | 6E, S1 |  |  |
| muscle 10 |  | The muscle that arises from the pronotum and inserts on the protrochantin. | 6C, 6E, S1 |  |  |
| muscle 11 | propleuro-procoxal muscle | [http://purl.obolibrary.org/obo/HAO_0000860](http://purl.obolibrary.org/obo/HAO_0000860;) | 6A, S1 | propleural ridge, apophysis, and anterior propleuron | anterior procoxal rim |
| muscle 12 |  | The muscle that arises from the pronotum and inserts on the prothoracic meron. | 6C, S1 |  |  |
| muscle 13 | propleuro-protrochanteral muscle | [http://purl.obolibrary.org/obo/HAO_0000861](http://purl.obolibrary.org/obo/HAO_0000861;) | 6E, S1 | lateral area of furco-pleural bridge and anterior propleuron | depressor tendon of protrochanter |
| muscle 14 | profurco-procoxal muscle | [http://purl.obolibrary.org/obo/HAO_0001891](http://purl.obolibrary.org/obo/HAO_0001891;) | 6E, S1 | medial area of furco-pleural bridge | posterolateral procoxal rim |
| muscle 15 | profurco-prophragmal muscle | <http://purl.obolibrary.org/obo/HAO_0001774> | 6C, S1 | apical dorsal profurcal arm | anterolateral prophragma |
| muscle 16 | anterior thoracic spiracle occlusor muscle | [http://purl.obolibrary.org/obo/HAO_0001115](http://purl.obolibrary.org/obo/HAO_0001115;) | 7F | prepectus | lids [1] of anterior thoracic spiracles |
| muscle 17 | prophragmo-postoccipital muscle | [http://purl.obolibrary.org/obo/HAO_0000333](http://purl.obolibrary.org/obo/HAO_0000333;) | 6C, 6D, S1 | anterolateral prophragma and anterior margin of mesonotum (anterior to prophragma) | median area of postocciput |
| muscle 18 | first mesopleuro-mesonotal muscle | [http://purl.obolibrary.org/obo/HAO_0000332](http://purl.obolibrary.org/obo/HAO_0000332;) | 2A |  |  |
| muscle 19 | prophragmo-mesophragmal muscle | [http://purl.obolibrary.org/obo/HAO_0000335](http://purl.obolibrary.org/obo/HAO_0000335;) | 2A, 7B, 7D |  |  |
| muscle 20 | mesonoto-mesolaterophragmal muscle | [http://purl.obolibrary.org/obo/HAO_0001643](http://purl.obolibrary.org/obo/HAO_0001643;) |  |  |  |
| muscle 22 | mesonoto-mesotrochantinal muscle | [http://purl.obolibrary.org/obo/HAO_0001361](http://purl.obolibrary.org/obo/HAO_0001361;) |  |  |  |
| muscle 23 | mesonoto-mesotrochanteral muscle | [http://purl.obolibrary.org/obo/HAO_0001894](http://purl.obolibrary.org/obo/HAO_0001894;) |  |  |  |
| muscle 24 | mesonoto-mesocoxal muscle | [http://purl.obolibrary.org/obo/HAO_0001644](http://purl.obolibrary.org/obo/HAO_0001644;) |  |  |  |
| muscle 25 | second mesopleuro-mesonotal muscle | [http://purl.obolibrary.org/obo/HAO_0000924](http://purl.obolibrary.org/obo/HAO_0000924;) |  |  |  |

**^1^**URIs resolve to definitions and included metadata from the supporting anatomy ontologies.

References

1. Bhatnagar BS (1974) Spiracles in certain terrestrial Heteroptera (Hemiptera). Int J Insect Morphol Embryol 1: 207–217. doi:10.1016/0020-7322(72)90028-1
